# Alpha‐linolenic acid given as an anti‐inflammatory agent in a mouse model of colonic inflammation

**DOI:** 10.1002/fsn3.1225

**Published:** 2019-11-19

**Authors:** Juan Wen, Israr Khan, Anping Li, Xinjun Chen, Pingrong Yang, Pingshun Song, Yaping Jing, Junshu Wei, Tuanjie Che, Chunjiang Zhang

**Affiliations:** ^1^ School of Life Sciences Lanzhou University Lanzhou China; ^2^ Key Laboratory of Cell Activities and Stress Adaptations Ministry of Education Lanzhou University Lanzhou China; ^3^ Gansu Key Laboratory of Biomonitoring and Bioremediation for Environmental Pollution Lanzhou University Lanzhou China; ^4^ Laboratory of Pathogenic Biology and Immunology Hainan Medical University Haikou China; ^5^ Gansu Institute of Drug Control Lanzhou China; ^6^ Gansu Key Laboratory of Functional Genomics and Molecular Diagnosis Lanzhou China

**Keywords:** inflammatory bowel disease, T‐cell‐related cytokines, α‐linolenic acid

## Abstract

This study examined the relationship between the high‐fat, high‐sugar diet (HFHSD) and trinitrobenzene sulfonic acid (TNBS) induced mouse colitis, the therapeutic effect of alpha‐linolenic acid (ALA) on mouse colitis, and the relationship between HFHSD and hyperlipidemia. We also examined the possible underlying mechanisms behind their interactions. Female BABL/c mice were fed with HFHSD for the 9 weeks. At the same time, ALA treatment (150 or 300 mg/kg) was administered on a daily basis. At the end of the 9 weeks, experimental colitis was induced by the intra‐colonic administration of TNBS. Body weight, spleen weight, disease activity index (DAI), histological changes, T‐cell‐related cytokine level, and lipid profiles were measured after treatment. TNBS induced severe clinical manifestations of colitis and histological damage. Low‐ALA (150 mg/kg) administration profoundly ameliorated TNBS‐induced clinical manifestations, body weight loss, spleen weight loss, and histological damage. On the contrary, the high‐ALA (300 mg/kg) administration did not ameliorate colitis and even exacerbated the symptoms. HFHSD consumption assisted TNBS in changing IL‐12, IFN‐γ, IL‐2, and IL‐17A in the liver. As expected, these changes were recovered through low‐ALA. In addition, HFHSD had a significant impact on the total cholesterol (TC), high‐density lipoprotein cholesterol (HDL‐C), and triglyceride (TG), which related to the increased risk of hyperlipidemia. In summation, HFHSD exacerbated the TNBS‐induced colitis via the Th1/Th17 pathway. The Low‐ALA (150 mg/kg) exhibited protective effects against the TNBS‐induced colitis via the Th1/Th2/Th17 pathway.

## Graphical abstract

High‐fat, high‐sugar diet (HFHSD) exacerbated the trinitrobenzene sulfonic acid (TNBS)‐induced colitis via the Th1/Th17 pathway activation. The Low‐alpha‐linolenic acid (ALA) exhibited protective effects against the TNBS‐induced colitis via the Th1/Th2/Th17 pathway suppression.

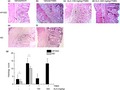

## 1 INTRODUCTION

Inflammatory bowel disease (IBD), including ulcerative colitis (UC) and Crohn's disease (CD), is chronic a remittent or progressive intestinal inflammatory conditions featuring by over‐expression of pro‐inflammatory cytokines and decreased levels of immunosuppressive cytokines (Kaser, Zeissig, & Blumberg, 2009; Niessner & Volk, 1995). Although a clear pathology of IBD has not been established, it is generally accepted that the course and development of IBD in the genetically susceptible host depend on the interaction of the immune system, intestinal microbiota, and environmental risk factors (Kaser et al., 2009). Numerous environmental factors such as diet, smoking, appendectomy, nonsteroidal anti‐inflammatory drugs, and antibiotics have been studied in relation to IBD (Molodecky & Kaplan, 2010). According to epidemiological investigations throughout the past decade, the incidence of IBD has doubled or tripled in several Asian countries. It is largely due to the increased consumption of the high‐fat, high‐sugar Western food, which contains high saturated fatty acid and n‐6 polyunsaturated fatty acids (n‐6 PUFAs) and low in n‐3 polyunsaturated fatty acids (n‐3 PUFAs; Manzel et al., 2014; Ng, 2015). The ratio of n‐6 to n‐3 PUFAs in the typical Western diet approaches 10:1 or even 25:1 (Palmquist, 2009; Poudyal, Panchal, Diwan, & Brown, 2011), whereas some researchers suggested ratio is 1:1 (Simopoulos, 2002).

The balance of n‐3 and n‐6 PUFAs plays a myriad important role in the human health. Moreover, their metabolism and inflammation cross‐regulate each other. Plant‐derived alpha‐linolenic acid (ALA; C18:3n‐3) and linoleic acid (LA; C18:2n‐6) are two essential fatty acids since they are not synthesized in the mammalian body, and thus obtained from plant sources diet (Baker, Miles, Burdge, Yaqoob, & Calder, 2016). They share a common metabolic pathway. ALA competes with LA in binding Δ6‐desaturase to divert metabolism toward more active n‐3 PUFA‐derived eicosapentaenoic acid (EPA; C20:5n‐3), docosapentaenoic acid (DPA; C22:5n‐3) and docosahexaenoic acid (DHA; C22:6n‐3), and less pro‐inflammatory arachidonic acid (AA; Bassaganya‐Riera & Hontecillas, 2010; Scorletti & Byrne, 2013). Then, EPA competes with AA as substrates of lipoxygenase (LOX) and cyclooxygenase (COX) to generate immunoregulator eicosanoids, including prostaglandins (PGs), thromboxanes, prostacyclins, and leukotrienes (LTs; Scorletti & Byrne, 2013). Compared with AA‐derived pro‐inflammatory eicosanoids, EPA‐derived eicosanoids have an anti‐inflammatory effect to re‐build immune homeostasis, for example, by reducing the expression of interleukins (Calder, 2013; Weaver et al., 2009). Recently, researchers found that n‐3 PUFAs can generate resolvins, protectins, and maresins induce pro‐resolving actions during an inflammatory response (Poudyal et al., 2011). Since IBD has an abnormal response of the intestinal immune system. Meanwhile, insufficient ingestion of n‐3 PUFAs is an environmental risk factor of IBD, and n‐3 PUFAs are known to have potent anti‐inflammatory and immunoregulatory properties (Bassaganya‐Riera & Hontecillas, 2010). It is imperative to investigate the potential beneficial effects of ensuring adequate intake n‐3 PUFAs or shifting the n‐6/n‐3 ratio on IBD.

Some encouraging studies showing that n‐3 PUFA‐rich diets exempted IBD in the clinic (Papadia et al., 2010) and animal models (Ibrahim et al., 2012; Monk et al., 2012). One study found that the transgenic mice rich in endogenous n‐3 PUFAs were protected from colitis (Hudert et al., 2006). However, the efficacy of n‐3 PUFAs as a form of complementary and alternative medicine (CAM) of IBD is still ambiguous, especially dose‐dependent actions of n‐3 PUFAs on IBD efficacy have not been well described (Calder, 2013). For instance, Hillary suggested to establishing a tolerable upper limit for DHA, regarding to the fact that dietary high‐dose‐DHA‐rich fish oil diet exacerbated mouse colitis (Woodworth et al., 2010). In addition, despite plant‐derived ALA is the major n‐3 fatty acid consumed in most human diets. Most of the related studies focused on marine‐derived EPA/DHA supplements or EPA/DHA‐rich fish oil (Calder, 2013). The remission efficacy of ALA‐rich flaxseed oil for IBD has been well documented, but the effectiveness of purified n‐3 PUFAs pathway primary fatty acid ALA is still less clear (Cohen, Moore, & Ward, 2005; Hassan et al., 2010; Monk et al., 2012). Therefore, it is worth investigating the effect of modifying n‐6/n‐3 ratio to provide reference to clinic practice. High‐fat, high‐sugar diet (HFHSD) also leads to hyperlipidemia and insulin resistance (IR), which may lead to coronary heart disease (CHD) (Munshi, Joshi, & Rane, 2014). Hyperlipidemia is characterized by a disorder of the total cholesterol (TC), low‐density lipoprotein cholesterol (LDL‐C), high‐density lipoprotein cholesterol (HDL‐C), and triglyceride (TG; Umar et al., 2015). More interestingly, the n‐3 PUFAs reduce the risk of this kind of diseases partly by improving the blood lipid profile (Poudyal et al., 2011). Given its potentially serious health effects, we are also interested in examining the risk of hyperlipidemia in the mouse colitis model.

In the present study, we investigate the effects of HFHSD and ALA supplement to trinitrobenzene sulfonic acid (TNBS)‐induced mouse colitis by a dietary intervention strategy, which is consistent with the current popular Western food. These experiments suggest that consuming a low dose of ALA daily could significantly prevent the incidence of colitis by the capacity of ALA and its metabolites to adjust the expression of T helper (Th) cell‐related cytokines in the colon. However, over‐increasing n‐3/n‐6 PUFAs ratio did not protect mice from TNBS‐induced colitis and could even exacerbate the symptoms.

## 2 MATERIALS AND METHODS

### 2.1 Animals

All animal studies were performed in accordance with protocols approved by the Ethics Committee of Animal Experiments of Lanzhou University. Female BALB/c mice, aged 4 weeks, were obtained from the Lanzhou Institute of Husbandry and Pharmaceutical Sciences of Chinese Academy of Agricultural Science (Lanzhou, China). The mice were housed in the plastic cages under the temperature and light‐controlled facility for 12‐hr light/dark cycle and were provided with free access to food and tap water. The mice were allowed to acclimate to these conditions for at least 7 days before inclusion in experiments.

### 2.2 Induction of colitis and experimental design

Unless indicated the mice were fed with purified 45% HFHSD (Trophic Animal Feed High‐tech Co., Ltd), and in which all nutritional requirements were met or exceeded (Table 1). In the meantime, the mice were gavaged with a 0.1 ml vehicle of ALA (Sigma‐Aldrich) solution daily for 9 weeks. For induction of colitis, the food was withdrawn overnight for 12 hr, and then, prior to the hapten, 0.1 ml of TNBS (2 mg in 50% ethanol; Sigma‐Aldrich) was administered intra‐rectally via a catheter 3 days before sacrifice. Control mice received 50% ethanol. The mice were divided into four groups (*n* = 9). ALA was dispersed in 0.25% Tween‐20 just before using to prevent oxidation. The four treatment groups were as follows: (a) vehicle/EtOH, (b) vehicle/TNBS, (c) ALA (150 mg kg^−1^ day^−1^)/TNBS, and (d) ALA (300 mg kg^−1^ day^−1^)/TNBS. Three days after TNBS administration, the mice were euthanized.

**Table 1:** Composition of experimental diet

Ingredient	g/kg
Casein	262.2
Corn starch	84.8
Maltodextrin	116.5
Sucrose	201.4
Cellulose	58.3
Lard oil	206.8
Soybean oil	29.1
Tert‐butylhydroquinone	0.05
Mineral mix AIN‐93G	35.2
Vitamin mix AIN‐93VX	0.84
l‐cysteine	3.5
Choline bitartrate (41.1% choline)	2.33

Following the TNBS challenge, the mice were examined for clinical colitis by measuring daily weight, daily hem‐occult positivity, and the presence of gross blood and stool consistency, and the disease activity index (DAI) was calculated according to the method used in our laboratory (Wen et al., 2015).

### 2.3 Histopathological analysis and histological scoring

The mice were sacrificed by cervical dislocation at the end of the experiment. A specimen from the distal third of the colon was fixed overnight with 4% phosphate‐buffered (pH 7.2) paraformaldehyde, then embedded in paraffin and cut longitudinally at 5 μm thickness, and stained with hematoxylin and eosin (H&E; Wen et al., 2016). Three sections per slide were scored on a blind basis, and histological analysis was performed on a blind basis as standard protocols.

### 2.4 Tissue lipid profile analysis

Total cholesterol, HDL cholesterol, LDL cholesterol, and triglyceride in the colon and in the liver were measured using the ELISA Kit (Beijing Beihua Clinical Reagent Co., Ltd).

### 2.5 Colon cytokine production analysis by LINCOplex assay

Concentrations of IL‐12, IFN‐γ, IL‐4, IL‐2, IL‐17A, TGF‐β, and IL‐10 in the colon homogenate were measured in duplicate by the LINCOplex assay (Suzhou SJ Biomaterials Co., Ltd.), a sandwich ELISA.

### 2.6 Statistical analysis

All results are expressed as mean values with their standard deviation for each group. Significant differences were carried out with Origin 2017 (OriginLab), and were established by one‐way ANOVA with post hoc Fisher's least significant difference (LSD) test for comparative analysis with vehicle/TNBS group unless otherwise indicated. *p* < .05 was regarded as statistically significant.

## 3 RESULTS

### 3.1 The effect of ALA on the clinical index of TNBS‐induced colitis mice

**Figure 1:**
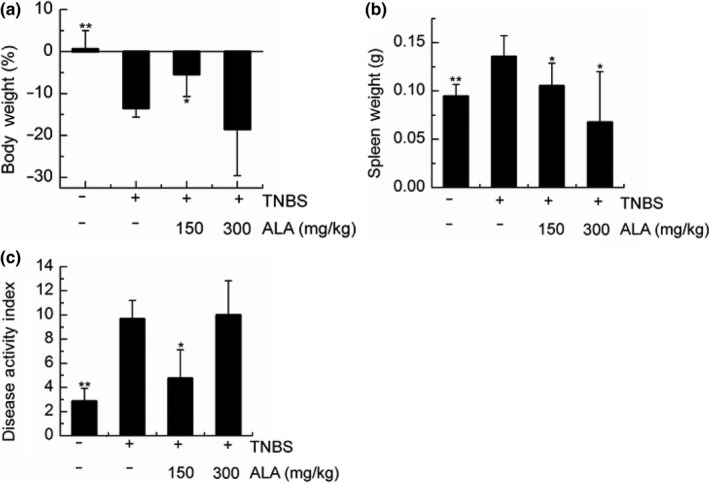
The effects of alpha‐linolenic acid (ALA) on the clinical index of trinitrobenzene sulfonic acid (TNBS)‐induced colitis mice. (a) Last 4 weeks body weight (% change), (b) spleen weight (g), and (c) disease activity index (DAI) at the day of sacrifice. Values are expressed as mean ± standard deviation, (*n* = 9). Significant difference was analyzed by comparing to the vehicle/TNBS group. *p < .05, **p < .01

In this paper, body weight, spleen weight, and DAI were regarded as a clinical index of TNBS‐induced colitis mice (Figure 1). Mice body weight was tracked for 4 weeks until the sacrifice. The body weight of the vehicle/EtOH increased slightly from 22.83 ± 2.05 g (5 weeks) to 22.96 ± 4.05 g (9 weeks) with daily consumption of HFHSD. Compared to the vehicle/TNBS group, the low‐ALA (150 mg/kg)/TNBS group had significantly higher body weight (Figure 1a). The vehicle/TNBS group had significantly higher spleen weight than the other three groups (Figure 1b), which meant there was a more active immune response. The DAI was determined by scoring changes in the body weight, hem‐occult positivity, or gross blood and stool consistency (Cooper, Murthy, Shah, & Sedergran, 1993). In our experiment, the vehicle/EtOH group and low‐ALA (150 mg/kg)/TNBS group had significantly lower the DAI than the vehicle/TNBS group, but the high‐ALA treatment did not reduce the DAI in the TNBS‐induced colitis (Figure 1c).

### 3.2 The effect of ALA and HFHSD on histopathological damage of TNBS‐induced colitis mice

**Figure 2:**
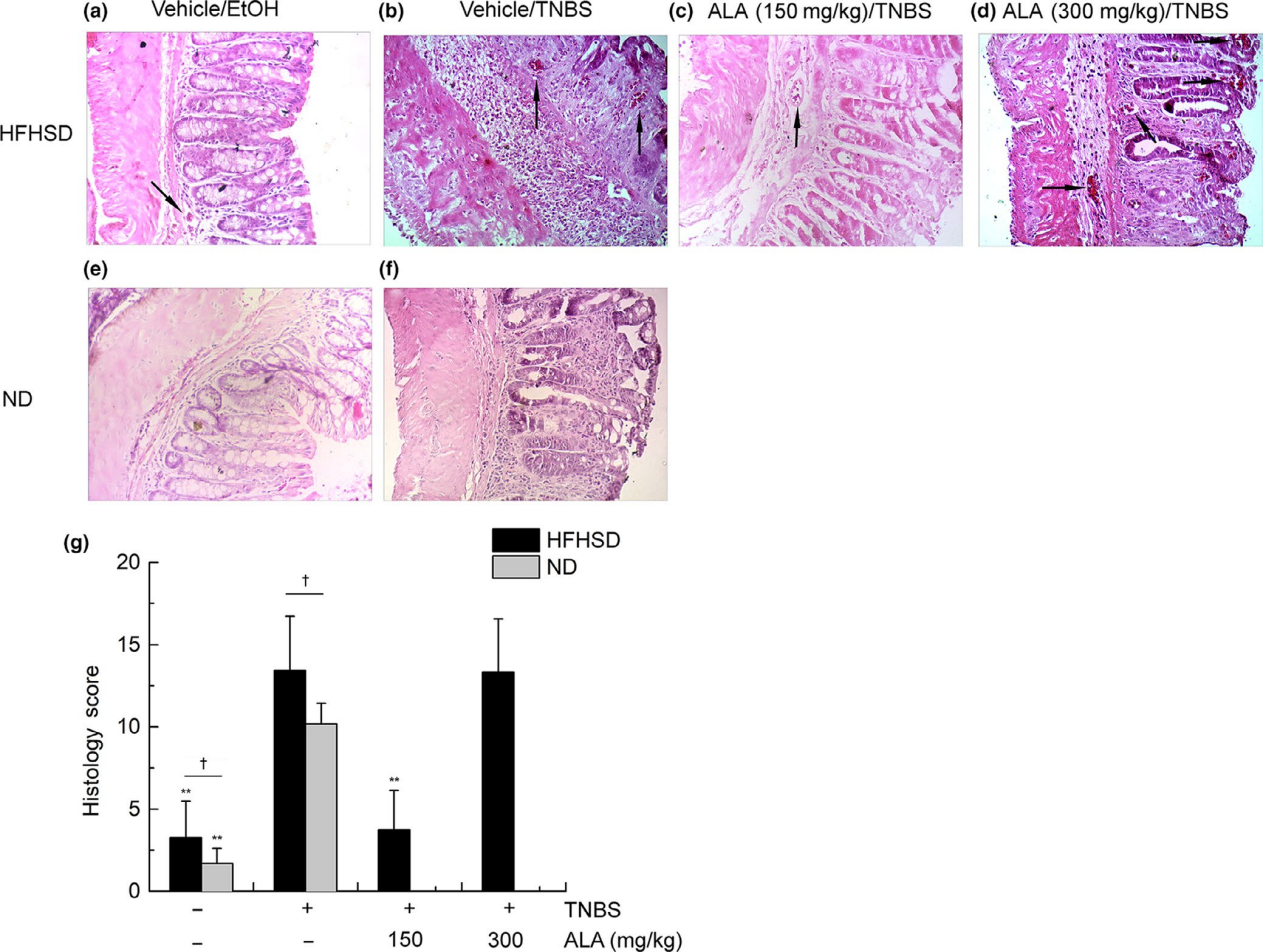
The effect of alpha‐linolenic acid (ALA) and high‐fat, high‐sugar diet (HFHSD) on colonic histology of trinitrobenzene sulfonic acid (TNBS)‐induced colitis mice. (a) Histological image (H&E stain) of vehicle/EtOH treatment group fed by HFHSD, (b) histological image (H&E stain) of vehicle/TNBS treatment group fed by HFHSD, (c) histological image (H&E stain) of low‐ALA/TNBS treatment group fed by HFHSD, and (d) histological image (H&E stain) of high‐ALA/TNBS treatment group fed by HFHSD. Black arrows in (a–d) indicate infiltration of inflammatory cells. (e) Histological image (H&E stain) of vehicle/EtOH treatment group fed by HFHSD, (f) histological image (H&E stain) of vehicle/TNBS treatment group fed by HFHSD, (g) histology scores. Values are expressed as mean ± standard deviation, (*n* = 9). Significant difference between HFHSD‐fed groups was analyzed by comparing to the vehicle/TNBS group, *p < .05, **p < .01. Besides, significant difference between different diet‐fed groups was analyzed by comparing between the same intrarectal administration groups, ^†^ *p* < .05

Trinitrobenzene sulfonic acid is a very strong chemical for inducing mouse colitis. The histopathological image of vehicle/TNBS mouse indicated that the overall surface epithelium was almost destroyed by the TNBS, and two obvious regions of inflammatory cell infiltration appeared in mouse colon (Figure 2b). Low‐ALA‐treated mice had a relatively intact surface epithelium and only one region of inflammatory cell infiltration (Figure 2c). However, a small amount of surface epithelium structure change and four regions of inflammatory cell infiltration were observed in the high‐ALA‐treated mouse colon (Figure 2d).

It was worth noting that one region of inflammatory cell infiltration was found in the vehicle/EtOH mouse colon, albeit with an intact surface epithelium (Figure 2a). Significant low scores were obtained by histological scoring in the vehicle/EtOH group and low‐ALA/TNBS group (Figure 2g). The histopathology (Figure 2e,f) and histology score (Figure 2g) of normal diet (ND) mice were also tested here.

### 3.3 The effect of ALA and HFHSD on hepatic and colonic lipid profile of TNBS‐induced colitis mice

**Figure 3:**
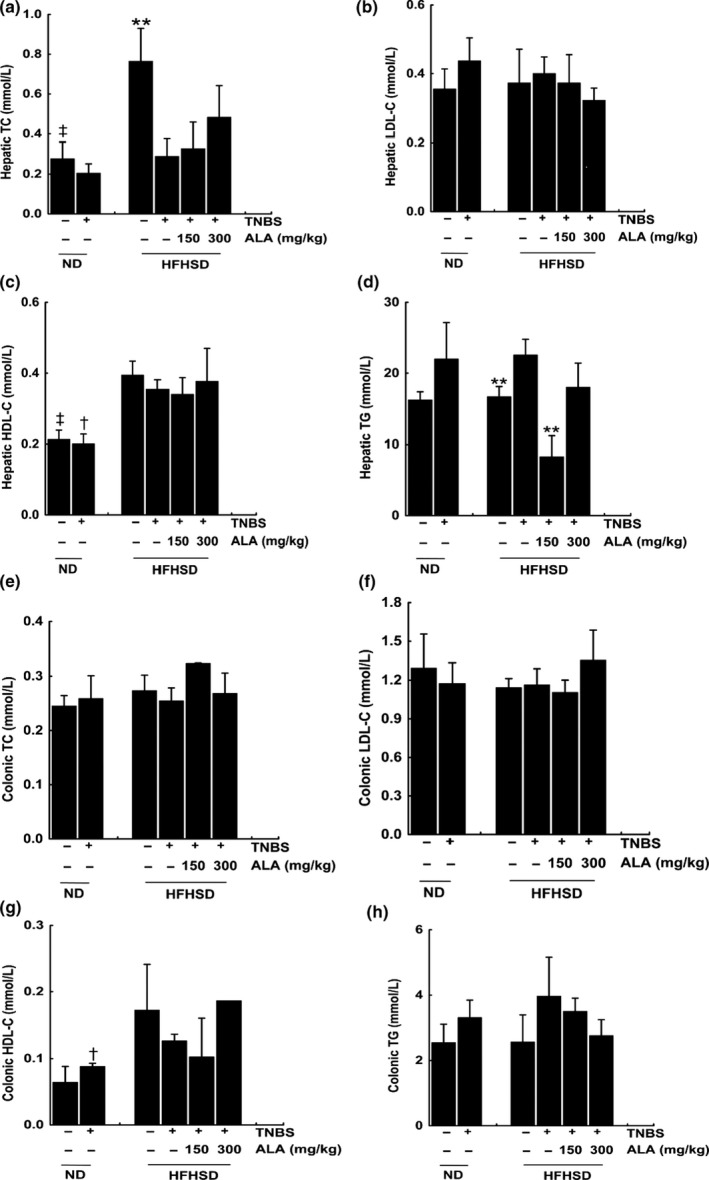
The effect of alpha‐linolenic acid (ALA) and high‐fat, high‐sugar diet (HFHSD) on hepatic and colonic lipid profile of trinitrobenzene sulfonic acid (TNBS)‐induced colitis mice. (a) Concentration of hepatic TC, (b) concentration of hepatic LDL‐C, (c) concentration of hepatic HDL‐C, (d) concentration of hepatic TG, (e) concentration of colonic TC, (f) concentration of colonic LDL‐C, (g) concentration of colonic HDL‐C, and (h) concentration of colonic TG. Values are expressed as mean ± standard deviation, (*n* = 9). Significant difference between HFHSD‐fed groups was analyzed by comparing to the vehicle/TNBS group. **p < .01. Besides, significant difference between different diet‐fed groups was analyzed by comparing between the same intrarectal administration groups, ^†^ *p* < .05, ^‡^ *p* < .01

Fed with HFHSD did not cause a significant decrease or an increase in the hepatic LDL‐C (Figure 3b), hepatic HDL‐C (Figure 3c), colonic TC (Figure 3e), colonic LDL‐C (Figure 3f), colonic HDL‐C (Figure 3g), or colonic TG (Figure 3h). These were in line with the TNBS‐treated mice decreasing body weight significantly (Figure 1a). Meanwhile, comparing to vehicle/TNBS‐treated group, hepatic TG of vehicle/EtOH group, and low‐ALA/TNBS group were decreased significantly (Figure 3d), while hepatic TC reduced only in vehicle/TNBS and low‐ALA‐treated group (Figure 3a). It is worth noting that elevated TG is one of the features of hyperlipidemia (Munshi et al., 2014). Inclusion, no significant changes of lipid profile were observed in all HFHSD mice groups.

### 3.4 The effect of ALA and HFHSD on clinical index of TNBS‐induced colitis mice

**Figure 4:**
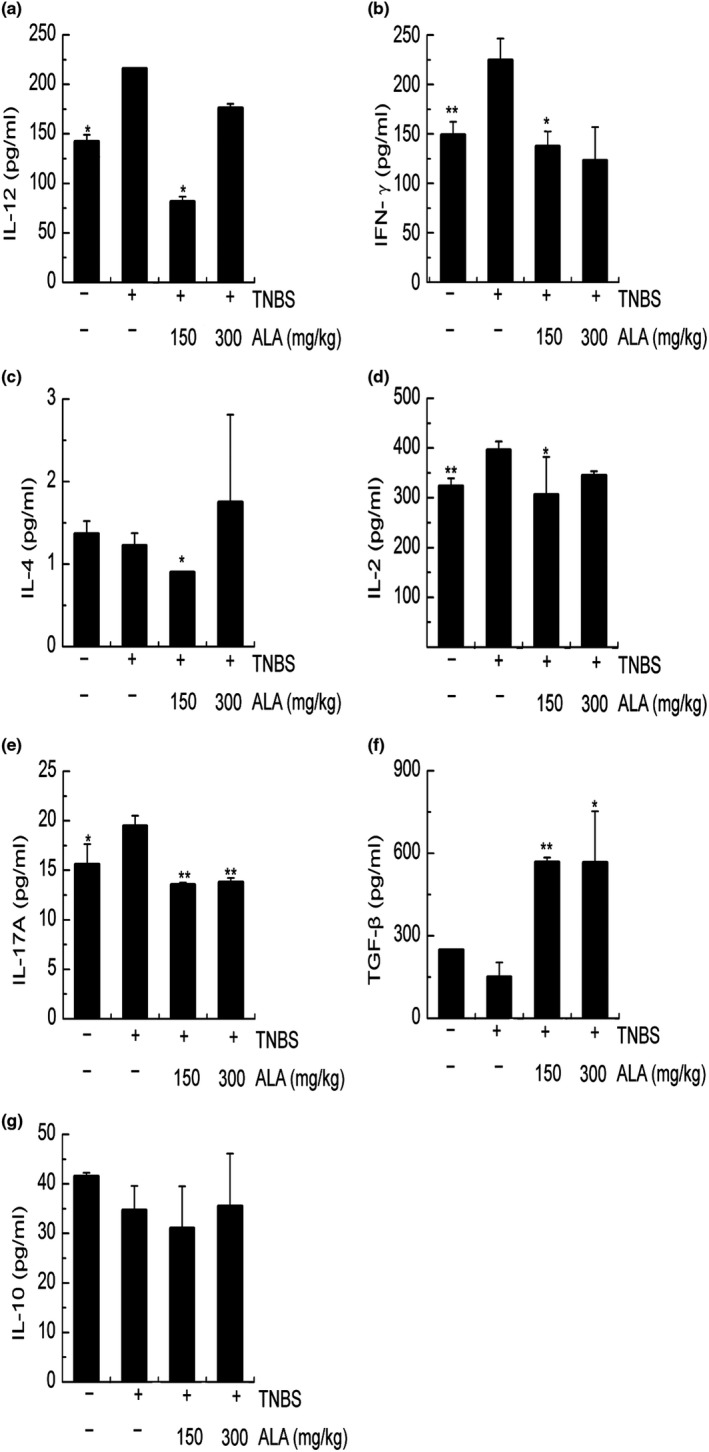
The effect of alpha‐linolenic acid (ALA) and high‐fat, high‐sugar diet (HFHSD) on colonic Th cell‐related cytokines of trinitrobenzene sulfonic acid (TNBS)‐induced colitis mice. (a) Concentration of colonic IL‐12, (b) concentration of colonic IFN‐γ, (c) concentration of colonic IL‐4, (d) concentration of colonic IL‐2, (e) concentration of colonic IL‐17A, (f) concentration of colonic TGF‐β, and (g) concentration of colonic IL‐10. Values are expressed as mean ± standard deviation, (*n* = 9). Significant difference was analyzed by comparing to the vehicle/TNBS group. *p < .05, **p < .01

In this study, Th1 cell‐related cytokines IL‐12 and IFN‐γ were found significantly lower in the vehicle/EtOH group and low‐ALA/TNBS group compared to vehicle/TNBS group (Figure 4a,b). Th2 cell‐related cytokines IL‐4 decreased significantly in the low‐ALA/TNBS group (Figure 4c). IL‐2 and IL‐17, Th17‐related cytokines, were also measured in current study. Both IL‐2 and IL‐17 were found significantly lower in the vehicle/EtOH group and low‐ALA/TNBS group; IL‐17 was also significantly lower in the high‐ALA/TNBS group (Figure 4d,e). TGF‐β increased significantly in both ALA treatment groups (Figure 4f). Finally, there were no significant differences in the IL‐10 concentration (Figure 4g).

## 4 DISCUSSION

As an important environmental factor in the IBD pathogenesis, daily diet, especially HFHSD Western food, has been studied extensively in both clinical and animal research. It has been widely accepted that the unbalanced n‐3 and n‐6 PUFAs in HFHSD contribute significantly to IBD pathogenesis, and our data support this. In the present study, mice were fed with HFHSD and were supplemented with ALA to balance the n‐3 and n‐6 PUFAs. We found that consuming a low dose of ALA daily could significantly remit TNBS‐induced colitis in mice via the Th1/Th2/Th17 pathway. However, over‐increasing n‐3/n‐6 PUFAs ratio did not protect mice from TNBS‐induced colitis and could even exacerbate the symptoms. A tolerable upper limit for ALA intake is needed to establish, particularly in the context of chronic inflammatory conditions such as IBD.

In the recent decades, the morbidity of IBD increased in Asian population largely due to consumption of the Western diet, that leading to an imbalance in the ratio of n‐6/n‐3 PUFAs, in an inclination of n‐6 PUFAs. The consumption of the diet containing high intake of n‐6 PUFAs and low level or insufficient n‐3 PUFAs leads in increasing the risk of IBD incidence (Bassaganya‐Riera & Hontecillas, 2010; Scaioli, Liverani, & Belluzzi, 2017). Meanwhile, epidemiological, human, animal, and cell culture studies demonstrated a positive relationship between consumption of n‐3 PUFAs and health benefits, such as reducing the inflammatory conditions in autoimmune disease and relieving symptoms of cardiovascular disease (Baker et al., 2016). Thus, optimizing the ratio of n‐3/n‐6 PUFAs in diets and ensuring adequate intake of n‐3 PUFAs as being treated as an important consideration. ALA, which has been absorbed from the gut, will metabolize to incorporate into cell membranes, energy production, pools for storage, and conversion to long‐chain n‐3 PUFAs (Baker et al., 2016). Due to the fact that n‐3 PUFAs family may regulate fuel partitioning, directing fatty acids from tissue storage to oxidation, they could potentially be exploited for such diverse effects as protection against obesity, hyperlipidemia, the metabolic syndrome (Baker et al., 2016; Palmquist, 2009). Current paper evaluated the effectiveness of purified ALA for colitis and hyperlipidemia by balancing the intake disproportionality of n‐6/n‐3 PUFAs.

Trinitrobenzene sulfonic acid‐induced colitis in the female BALB/c mice was applied to study the function of ALA in this experiment. The observation that the last 4 weeks body weight of HFHSD‐fed mice without colitis (vehicle/EtOH group) showed no substantial increase (Figure 1a) was likely due to the BALB/c mouse is a very stable mouse strain. However, we found that the hepatic TC and hepatic HDL‐C of the HFHSD‐fed vehicle/EtOH mice were significantly higher than in the normal diet‐fed vehicle/EtOH mice (Figure 3a,c). These results suggest that the HFHSD increases the risk of hyperlipidemia, even if the body weight does not change much.

As mentioned previously, increased in the lipid profile is a characteristic of hyperlipidemia. In our study, no significant differences were found in the colonic lipid profiles (Figure 3e,h), hepatic LDL‐C (Figure 3b), or hepatic HDL‐C (Figure 3c) in different ALA‐treated groups compared to the vehicle/TNBS group. The current results still do not indicate whether ALA has benefit for hyperlipidemia. Further relative study could display without the interference of experimental colitis.

High‐fat, high‐sugar diet also has a significant contribution in the mouse colitis. The histology score (Figure 2g) was significantly higher in the HFHSD‐fed vehicle/TNBS mice than in the normal diet vehicle/TNBS mice. In addition, infiltration of inflammatory cells was found in the HFHSD‐fed vehicle/TNBS mouse colon (Figure 2a), but not in the normal diet vehicle/EtOH mice (Figure 2e), indicating that HFHSD causes low‐grade inflammation.

Alpha‐linolenic acid, EPA, and DHA are grouped together as well‐studied n‐3 PUFAs. Evidence suggests that human as well as rodents among other mammals, do not efficiently convert medium‐chain ALA to long‐chain EPA or DHA, most studies focused on EPA and DHA (Poudyal et al., 2011). However, we still keep interesting in ALA due to following reasons. First, studies proved that ALA‐rich oil could be a beneficial functional food on IBD (Cohen et al., 2005; Hassan et al., 2010; Poudyal, Panchal, Ward, & Brown, 2013). Secondly, the dietary intake of ALA is much higher than EPA and DHA among people who do not regularly consume oily fish and consume ALA‐rich flaxseed oil daily (Baker et al., 2016). Thirdly, unlike men do not efficiently convert ALA to EPA and DHA, women possess a higher capacity for ALA conversion (Baker et al., 2016). In young women, estimated net fractional ALA inter‐conversion was EPA 21%, DPA 6%, and DHA 9% (Burdge & Wootton, 2002). In addition, model animals, like rats and mice, can also convert ALA to EPA and DHA (Scott & Bazan, 1989; Sinclair, Attar‐Bashi, & Li, 2002). Lastly, it also suggests that ALA has extra physiological responses, not relying on its metabolism to DHA and EPA. For instance, as mentioned before ALA competes with LA for the same metabolic pathway and so will result in the reduction of the AA content in the tissues, which might be important for immunoregulation (Baker et al., 2016; Calder, 2013). In this way, it is interesting to investigate the mechanisms of plant‐derived n‐3 PUFA, ALA for influencing IBD future. The present work focused on the ALA's immunoregulation function on TNBS‐induced colitis.

In the pathogenesis of the onset and development of IBD, mucosal immunity and the interactions between intestinal bacteria, environmental factors, and genetic factors play central roles (Yamada et al., 2016). Abundant studies demonstrated a direct connection between colitis and an abnormal response of Th cells. The maturation of Th cells could be summarized as following: Signal transducer and activator of transcription 4 (STAT4) is a transcription factor belonging to the STAT protein family, can be activated by interleukin (IL)‐12 and drives naïve CD4 cells to become IFN‐γ‐producing Th1 cells. IL‐4 can drive naïve CD4 cells to become IL‐4‐producing Th2 cells via signals through STAT6. TGF‐β, IL‐6, IL‐2, and IL‐1 can drive naive CD4 cells to become IL‐17‐producing Th17 cells via signals through RORγt. Finally, in the presence of IL‐2 and high concentration of TGF‐β, naive CD4 cells can develop into IL‐10 and TGF‐β production Treg‐β cells (Vojdani & Lambert, 2011). A great deal of research has been done on the role of mucosal Th1/Th2 balance in IBD. More recently, researchers have also focused on the Th17 and Treg (Leppkes et al., 2009; Round & Mazmanian, 2010). There are growing evidences that Th17‐related cytokine IL‐17 has a highly pathogenic role in the IBD pathogenesis, while Treg‐related cytokine IL‐10 is an immunosuppressive cytokine that protects humans and mice from IBD (Geuking et al., 2011; Leppkes et al., 2009; Round & Mazmanian, 2010). For instance, Calcitriol, a promising new therapeutic option for colitis, can change the Th1, Th2, Th17 and Treg cell profile (Daniel, Sartory, Zahn, Radeke, & Stein, 2008). A previous study in our laboratory shown that Bawei Xileisan (BXS), a traditional Chinese compound medicine, is curative in the DSS‐induced colitis by disruption of the Th17 pathway and the induction of a Th17/Treg imbalance (Wen et al., 2016).

In order to understand the overall effect of ALA on Th cells, we studied seven cytokines related Th1, Th2, Th17, and Treg cells in our experiment. Diets in the current study mimicked 2 g/day (low‐ALA) and 4 g/day (high‐ALA) human ALA consumption. Our results suggest that low‐ALA has protective effects against TNBS‐induced colitis via the Th1/Th2/Th17 pathway. The decrease of IL‐17 and the increase of TGF‐β were observed in the high‐ALA (300 mg/kg) group, whereas IL‐2 was not significantly changed in this group. Considering that the balance of the Th17/Treg will shift to Treg cells by signals from IL‐2 and high concentration of TGF‐β, high‐dose ALA might disrupt Th17 pathway in the TNBS‐induced colitis. Furthermore, Treg pathway was not influenced by high‐dose ALA supplement, because no significant difference of IL‐10 was observed.

Taking together, these results support the need to establish a tolerable upper limit for ALA intake, particularly in the context of chronic inflammatory conditions such as IBD. Considering the conversion of ALA to DHA is more on experimental animals than in humans (Baker et al., 2016). It still needs further investigation in the clinic.

## CONFLICT OF INTEREST

The authors declare that they do not have any conflict of interest.

## ETHICAL APPROVAL

This study was approved by the Animal Care and Ethics committee of the Lanzhou University.
